# Artificial intelligence automatic measurement technology of lumbosacral radiographic parameters

**DOI:** 10.3389/fbioe.2024.1404058

**Published:** 2024-07-01

**Authors:** Shuo Yuan, Ruiyuan Chen, Xingyu Liu, Tianyi Wang, Aobo Wang, Ning Fan, Peng Du, Yu Xi, Zhao Gu, Yiling Zhang, Lei Zang

**Affiliations:** ^1^ Department of Orthopedics, Beijing Chaoyang Hospital, Capital Medical University, Beijing, China; ^2^ School of Life Sciences, Tsinghua University, Beijing, China; ^3^ Department of Biomedical Engineering, School of Medicine, Tsinghua University, Beijing, China; ^4^ Longwood Valley Medical Technology Co., Ltd., Beijing, China

**Keywords:** artificial intelligence, deep learning, automatic measurement, lateral lumbar radiograph, lumbosacral radiographic parameters

## Abstract

**Background:**

Currently, manual measurement of lumbosacral radiological parameters is time-consuming and laborious, and inevitably produces considerable variability. This study aimed to develop and evaluate a deep learning-based model for automatically measuring lumbosacral radiographic parameters on lateral lumbar radiographs.

**Methods:**

We retrospectively collected 1,240 lateral lumbar radiographs to train the model. The included images were randomly divided into training, validation, and test sets in a ratio of approximately 8:1:1 for model training, fine-tuning, and performance evaluation, respectively. The parameters measured in this study were lumbar lordosis (LL), sacral horizontal angle (SHA), intervertebral space angle (ISA) at L4–L5 and L5–S1 segments, and the percentage of lumbar spondylolisthesis (PLS) at L4–L5 and L5–S1 segments. The model identified key points using image segmentation results and calculated measurements. The average results of key points annotated by the three spine surgeons were used as the reference standard. The model’s performance was evaluated using the percentage of correct key points (PCK), intra-class correlation coefficient (ICC), Pearson correlation coefficient (r), mean absolute error (MAE), root mean square error (RMSE), and box plots.

**Results:**

The model’s mean differences from the reference standard for LL, SHA, ISA (L4–L5), ISA (L5–S1), PLS (L4–L5), and PLS (L5–S1) were 1.69°, 1.36°, 1.55°, 1.90°, 1.60%, and 2.43%, respectively. When compared with the reference standard, the measurements of the model had better correlation and consistency (LL, SHA, and ISA: ICC = 0.91–0.97, r = 0.91–0.96, MAE = 1.89–2.47, RMSE = 2.32–3.12; PLS: ICC = 0.90–0.92, r = 0.90–0.91, MAE = 1.95–2.93, RMSE = 2.52–3.70), and the differences between them were not statistically significant (*p* > 0.05).

**Conclusion:**

The model developed in this study could correctly identify key vertebral points on lateral lumbar radiographs and automatically calculate lumbosacral radiographic parameters. The measurement results of the model had good consistency and reliability compared to manual measurements. With additional training and optimization, this technology holds promise for future measurements in clinical practice and analysis of large datasets.

## 1 Introduction

Low back pain is a common clinical symptom of lumbar diseases, affecting the quality of life and health of patients of all ages while imposing significant economic burdens on individuals, families, and governments (Hoy et al., 2012; Hong et al., 2013; Maher et al., 2017; Kim et al., 2019). According to research, low back pain is associated with lumbosacral instability (Panjabi, 2003). While many studies have focused on the treatment and prevention of lumbar diseases, there has been a shift toward researching the role of the lumbosacral sagittal alignment in lower back pain and lumbar diseases, emphasizing the importance of accurately measuring lumbosacral radiographic parameters (Kalidindi et al., 2022; Tartara et al., 2023).

The presence of various spinal disorders is linked to abnormalities in the spine’s sagittal alignment, making proper spinal sagittal alignment critical for quantitatively assessing spinal health (Scheer et al., 2013; Schwab et al., 2013; Liu et al., 2015; Brink et al., 2017). Spinal instability and the resulting compensatory reactions can put additional strain on important spinal structures, resulting in pain (Diebo et al., 2015; Lafage et al., 2017). Therefore, achieving proper sagittal plane alignment is a crucial treatment goal for spine surgeons. Accurately assessing and quantifying changes in lumbosacral radiographic parameters is critical for clinical diagnosis, treatment, surgical planning, and postoperative analysis of spinal diseases (Kumar et al., 2001; Schwab et al., 2010). However, current manual measurement method is time-consuming, rely on physician experience, and are susceptible to inter-observer and intra-observer variability, resulting in significant measurement errors that have an impact on clinical diagnosis and decision-making (Loder et al., 2004; Dang et al., 2005; Chan et al., 2014). Obviously, the traditional manual measurement method has failed to keep up with the advancement of imaging technology and the increase in the number of imaging examinations, making it difficult to meet the demand for accurate clinical diagnosis and treatment.

With the recent rapid development of artificial intelligence and its increasing integration into the field of orthopedics, using artificial intelligence technology to accurately process complex X-ray image data has emerged as a research trend (Wang et al., 2022). Recently, many studies have used models based on deep learning algorithms to measure various spinal parameters, improving the accuracy and speed of medical images analysis (McBee et al., 2018).

The goal of this study was to develop a deep learning-based model for automatically measuring lumbosacral radiographic parameters on lateral lumbar radiographs. Furthermore, this study will evaluate the performance of the model, which is expected to be an effective tool for replacing manual measurements if it achieves high accuracy and efficiency in measuring lumbosacral radiographic parameters.

## 2 Materials and methods

### 2.1 Date preparation

We retrospectively collected data from 2,853 patients who received standing lateral lumbar radiographs at the orthopedics outpatient department of Beijing Chaoyang Hospital between October 2022 and October 2023. The inclusion criteria were adult patients with vertebral endplate closure. The following exclusion criteria were used (Maher et al., 2017): a history of spinal surgery (Kim et al., 2019); severe spinal deformity (Hong et al., 2013); patients with metabolic bone disease, spinal fracture, tuberculosis, and tumors; and (Hoy et al., 2012) patients with poor X-ray image quality, severe osteophyte formation, or other factors affecting measurements. The hospital’s institutional review board and ethics committee approved this study. Furthermore, all aspects of this study conformed to the principles outlined in the Declaration of Helsinki.

**FIGURE 1:**
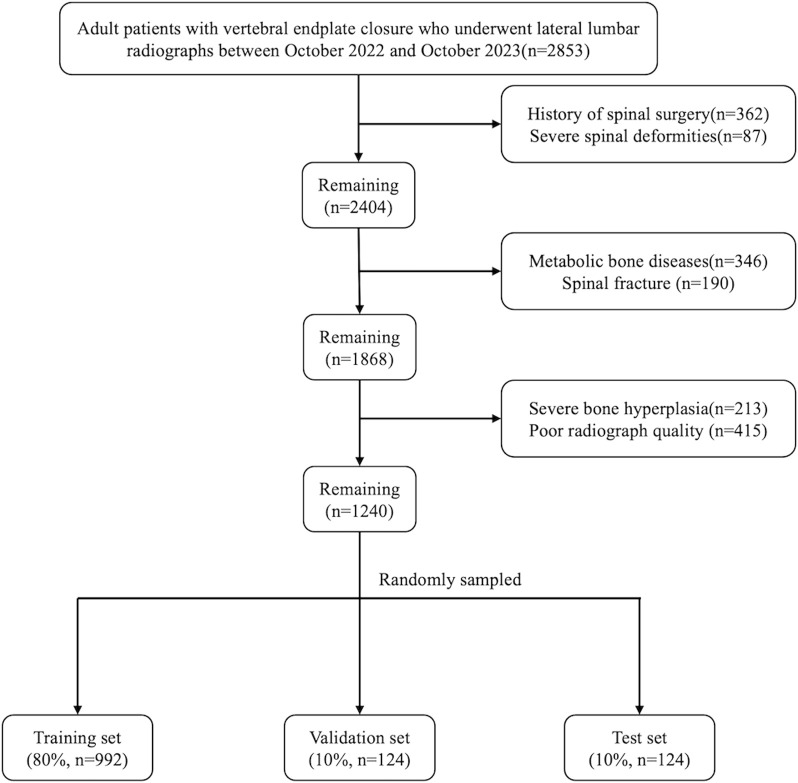
Flowchart of the process of selecting images.

A total of 1,240 lateral lumbar radiographs were included after screening based on the inclusion and exclusion criteria. These images were randomly divided into the training set, validation set and test sets in the ratio of 8:1:1. The test set (n = 124) was used to evaluate the final prediction performance of the model; the training set (n = 992) was used to train the model and optimize the model parameters; and the validation set (n = 124) was used to adjust the model hyperparameters and conduct a preliminary evaluation of the model performance. Figure 1 shows the flowchart of image screening.

### 2.2 Key point annotations

**FIGURE 2:**
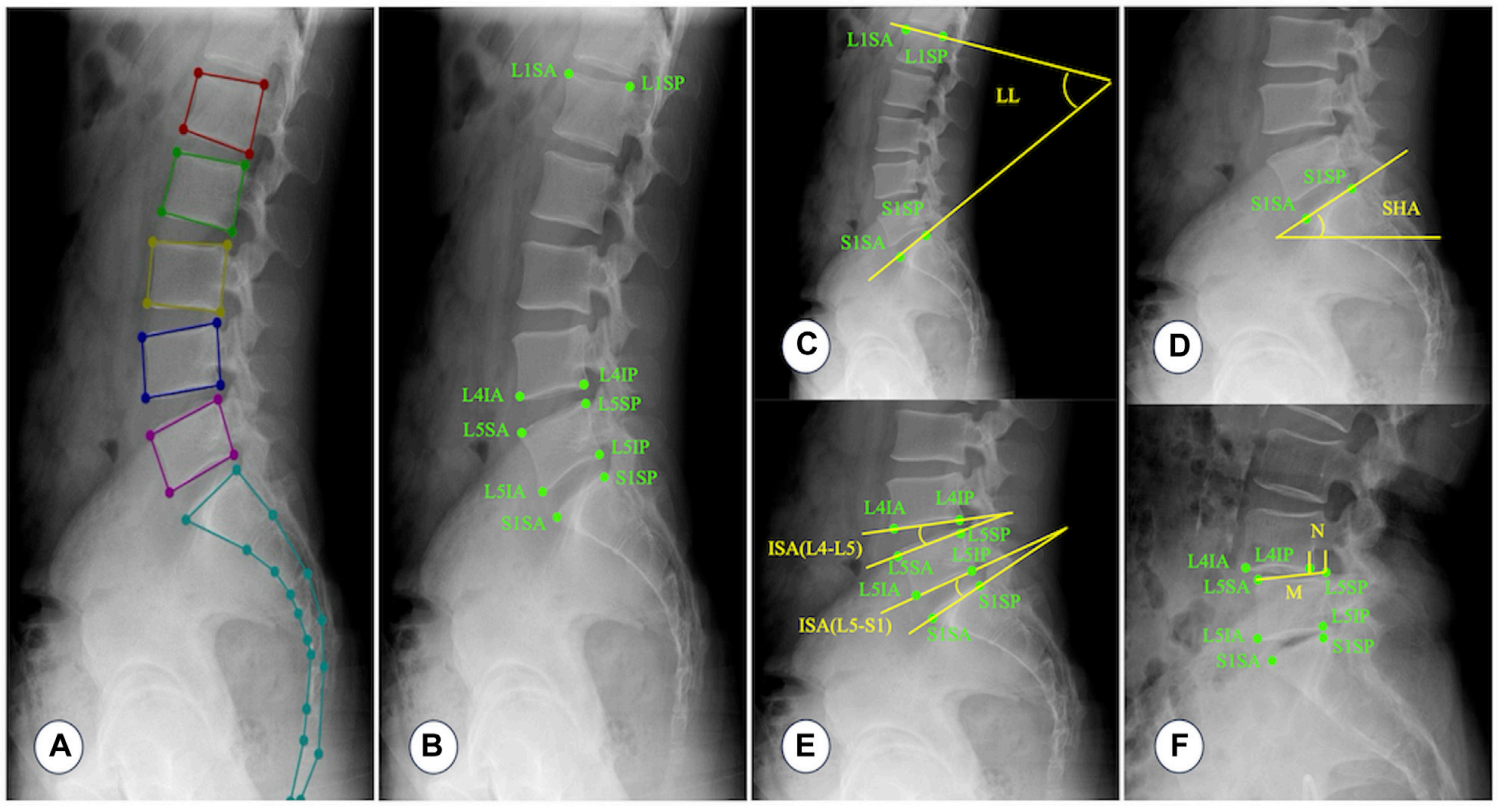
Annotating key points, naming vertebral key points, and measuring parameter values. **(A)** A typical example image annotated with Labelme software. **(B)** L1SA: Vertex of the anterior superior edge of L1 vertebra; L1SP: Vertex of the posterior superior edge of L1 vertebra; L4IA: Vertex of the anterior inferior edge of L4 vertebra; L4IP: Vertex of the posterior inferior edge of L4 vertebra; L5SA: Vertex of the anterior superior edge of L5 vertebra; L5SP: Vertex of the posterior superior edge of L5 vertebra; L5IA: Vertex of the anterior inferior edge of L5 vertebra; L5IP: Vertex of the posterior inferior edge of L5 vertebra; S1SA: Vertex of the anterior superior edge of S1 vertebra; S1SP: Vertex of the posterior superior edge of S1 vertebra. **(C)** Lumbar lordosis (LL): The angle between the tangent line of the upper endplate of the L1 vertebra and the tangent line of the upper endplate of the S1 vertebra. **(D)** Sacral horizontal angle (SHA): The angle between the tangent line of the upper endplate of the S1 vertebra and the horizontal line. **(E)** Intervertebral space angle (ISA): The angle between the tangent line of the lower endplate of the upper vertebra and the tangent line of the upper endplate of the lower vertebra. **(F)** Percentage of lumbar spondylolisthesis (PLS): Measure the distance (N) between the extension line of the posterior edge of the upper vertebra and the extension line of the posterior edge of the lower vertebra, and then measure the distance (M) between two points on the anterior and posterior edges of the upper endplate of the lower vertebra. Percentage of lumbar spondylolisthesis = N/M × 100%. Because lumbar instability is most common at the L4–L5 and L5–S1 segments, this study only measures ISA and PLS at the L4–L5 and L5–S1 segments.

Three spine surgeons (S1, S2, and S3) received standardized professional training and annotated 1,240 lateral lumbar radiographs using the Labelme software for model training and validation. Figure 2 depicts the detailed process of annotating key points, naming vertebral key points, and measuring parameter values (Koslosky and Gendelberg, 2020; Morita et al., 2020; Zhou et al., 2022). All images were annotated independently by each spine surgeon, with no knowledge of the annotations of the others. The test set was re-annotated by the spine surgeon (S1) after 1 month to evaluate intra-observer reliability.

### 2.3 Training model

**FIGURE 3:**
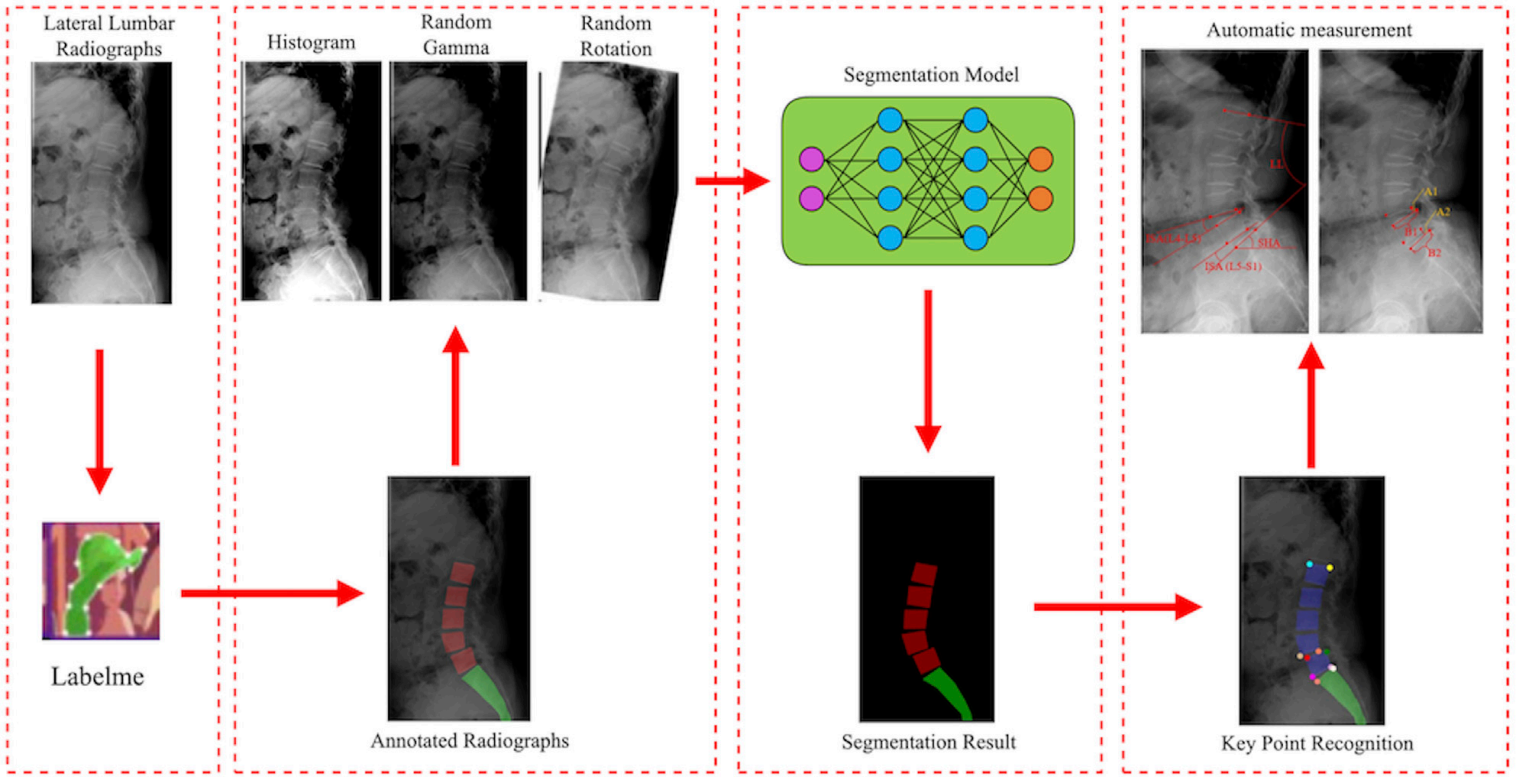
Flowchart of the model training.

First, lateral lumbar radiographs were annotated with the Labelme software. The model was then trained with the segmentation network model based on RADMFNet algorithm (described in greater detail later in Section 2.4 of the article). Histogram enhancement, random Gamma transformation, and random rotation of the original and annotated images were used to increase the number of samples and improve the robustness of the model (Shin et al., 2020). Finally, the corner detection algorithm was used to process the segmentation results and identify the corresponding key points. The model then computed LL, SHA, ISA(L4–L5), ISA(L5–S1), PLS(L4–L5), and PLS(L4–L5) using the positions of these key points. The training process of the model is shown in Figure 3.

### 2.4 Segmentation network

**FIGURE 4:**
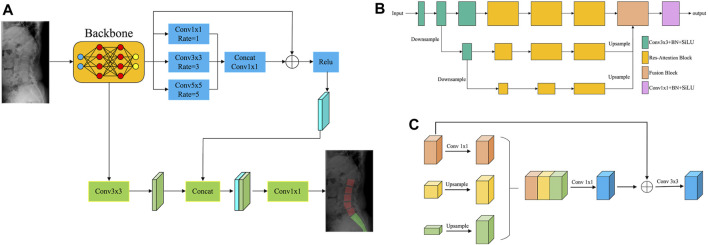
The segmentation network model. **(A)** Structure of the segmentation network. **(B)** Structure of the backbone network based on the multi-scale attention mechanism. **(C)** Network structure of the fusion module.

To create a fast and accurate segmentation network model, this study used dilated convolution (Zhang et al., 2020), ResNet (Tian et al., 2022), attention mechanism (Rondinella et al., 2023), multi-scale feature fusion (Gao et al., 2023), and other technologies. Figure 4A illustrates the structure of the segmentation network model.

First, the images were fed into the backbone network, which used the multi-scale attention mechanism to extract image features. Figure 4B shows the structure of the backbone network based on the multi-scale attention mechanism. The backbone network consisted of three distinct scale branches. Among them, the 3 × 3 Convolution + BN + SiLU module was utilized for feature extraction. The Res–Attention Block was primarily improved based on the SE–Resnet structure. The two consecutive FC layers (fully connected layers) of SE–Resnet were converted to 1 × 1 convolutions, significantly increasing the training efficiency of the network. Meanwhile, the activation function was changed from ReLU to SiLU, increasing the sensitivity of the model to edge information. The fusion module fused the feature maps from multiple scales, reducing the risk of overfitting and improving the generalization ability of the model. Figure 4C depicts the network structure of the fusion module. First, the multi-scale feature maps were unified to the same scale using the sampling operation. The feature maps were then concatenated, and 1 × 1 convolution was used to perform a preliminary fusion of inter-channel information. To ensure the integrity of detailed information, the preliminary fusion result was added to the feature map of the minimum scale and subjected to 3 × 3 convolution operations, effectively completing the feature fusion.

Next, the feature maps of the backbone network were processed by two branches: the Residual-based Dilated Convolutional Module and the Convolutional Feature Extraction Module. The Residual-based Dilated Convolutional Module must perform three dilated convolution operations on the feature maps: 1 × 1, 3 × 3, and 5 × 5. Dilated convolution broadened the receptive field, allowing for a more comprehensive perception of image features. To achieve a more powerful feature expression, the feature maps processed by each dilated convolution operation were concatenated and then subjected to a 1 × 1 convolution operation to fuse the features. Meanwhile, the information on the feature maps of the backbone network was better preserved by referencing the residuals, which avoided problems like gradient vanishing and improved the robustness of the model. The Convolutional Feature Extraction Module primarily re-extracted the image features obtained by the backbone network. It then combined the re-extracted image features with the feature maps from the Residual-based Dilated Convolutional Module to generate the segmentation result.

### 2.5 Identifying key points

**FIGURE 5:**
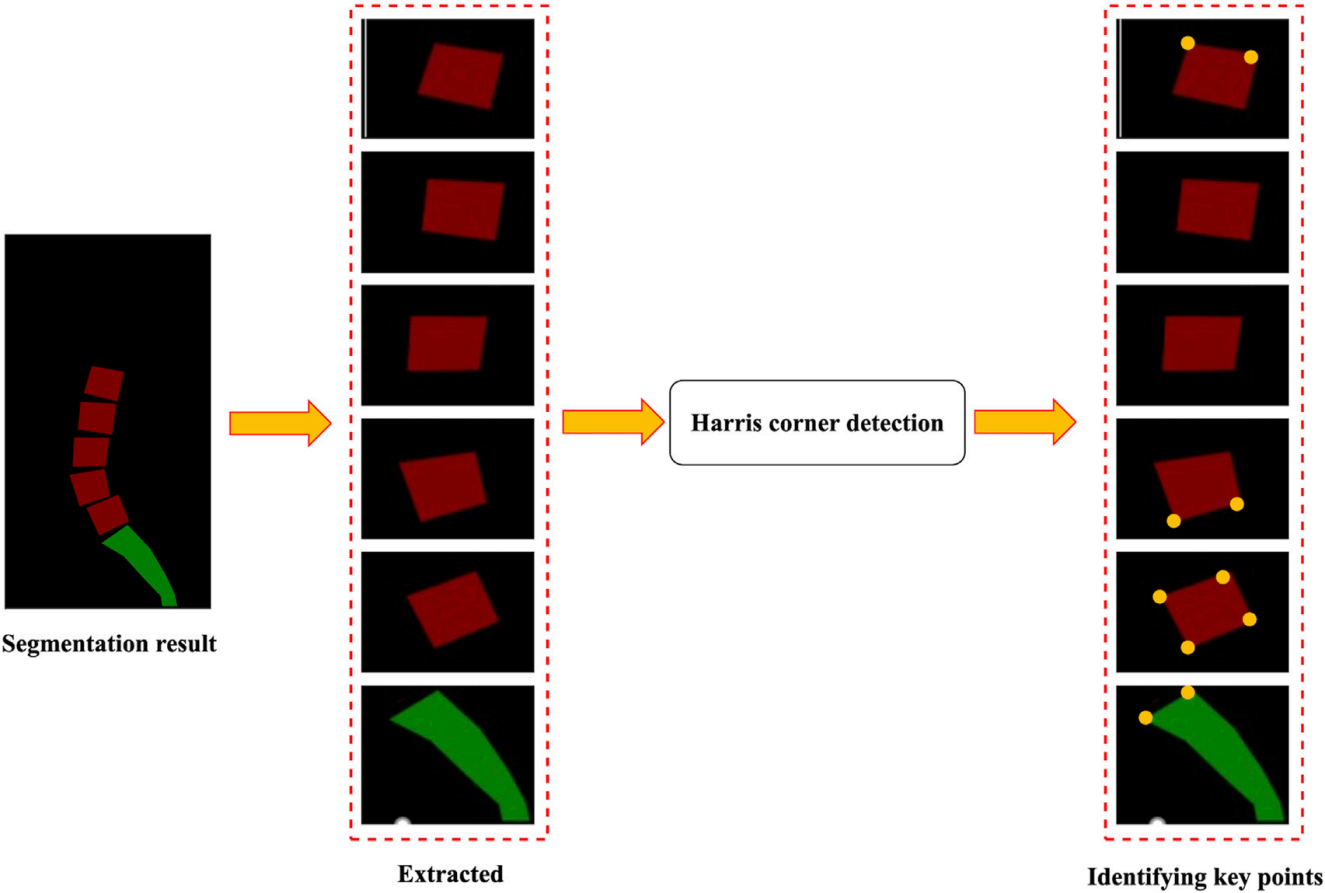
Flowchart for identifying key points.

The identification of key points was based on the segmentation results of the images. Figure 5 illustrates a flowchart for identifying key points. First, the lumbar vertebra and sacrum were extracted from the segmentation results by connecting regions. The Harris corner detection method was then applied to identify key points (Harris and Stephens, 1988). Harris corner detection was used to detect corners in images. First, this algorithm calculated the gradient at each pixel in images and the degree of gradient change in the neighborhood around each pixel. Then, it evaluated the direction and intensity of grayscale changes at each pixel by computing the covariance matrix. Based on the eigenvalues of the covariance matrix, the algorithm calculated the corner response function to determine whether the pixel was a corner. Finally, the pixel with the largest response value was selected by the method of non-maximum suppression to obtain the final corner point detection result.

### 2.6 Statistical analysis

SPSS (version 27.00; Chicago, Illinois, United States) was used for statistical analysis. The distribution of the demographic information of the patients across the training set, validation set, and test sets was represented with descriptive statistics. A *p* < 0.05 was considered statistically significant.

#### 2.6.1 Reliability of the key point annotations

To evaluate inter-observer and intra-observer reliability of key point annotations, percentages of key points within distance thresholds of 1, 2, 3, 4, and 5 mm were calculated.

#### 2.6.2 Segmentation performance

The accuracy and Dice coefficient were used to evaluate the segmentation performance of the model. The segmentation results for the lumbar and sacral regions were evaluated separately. We further compared the segmentation performance of our model with four other existing models, including UNet, Att-UNet, UNet 3+, and TransUNet. All models were trained on the same dataset.

#### 2.6.3 Performance of key point prediction

The performance of the model in predicting key points was evaluated using the PCK. The average of the results annotated by three spine surgeons was the reference standard. PCK denoted the percentage of predicted key points that were within a radius r of the reference standard.

#### 2.6.4 Measurement performance of the model

We compared and evaluated the measured values of the model with the average measured values of the three spine surgeons. We calculated the intra-class correlation coefficient (ICC), Pearson correlation coefficient (r), mean difference (MD), standard deviation (SD), mean absolute error (MAE), and root mean square error (RMSE) between the reference standard and model estimates to evaluate the performance of the model. ICC is an indicator of consistency, and ICC ≥ 0.75 is deemed sufficiently reliable. |r| ≥ 0.7 indicates a strong correlation. Additionally, box plots were used to show the distribution of error values between the measured values of the model and reference standard.

## 3 Results

### 3.1 Demographic data

We obtained 1,240 lateral lumbar radiographs (male-to-female ratio of 1:1). They were divided into three sets: 80% for training, 10% for validation, and 10% for test. The included data sets showed no significant differences in gender composition or age. The Demographic data distribution is shown in Table 1.

**TABLE 1:** Demographic characteristics of the patients.

Characteristic	Training set	Validation set	Test set
Number	992(80)	124(10)	124(10)
Age(year)*	52.46 ± 16.54	50.85 ± 16.16	51.79 ± 17.23
Sex			
Male	493(49.7)	59(47.6)	68(54.8)
Female	499(50.3)	65(52.4)	56(45.2)

Data are expressed as the number of patients, with percentages are in parentheses. * Data is expressed as mean ± SD.

### 3.2 Reliability of the key point annotations

The percentage of intra-observer annotation distance within the 3 mm threshold was 96%, while the inter-observer annotation distance within the 3 mm threshold was 83% (S1 and S2), 81% (S1 and S3), and 80% (S2 and S3), respectively (Table 2).

**TABLE 2:** Intra-observer and inter-observer reliability (%) for key point annotations.

Threshold(mm)	1	2	3	4	5
Intra-observer reliability	80	91	96	98	100
Inter-observer reliability					
S1 vs. S2	28	65	83	91	94
S1 vs. S3	26	64	81	88	93
S2 vs. S3	24	61	80	86	91

### 3.3 Segmentation performance

**FIGURE 6:**
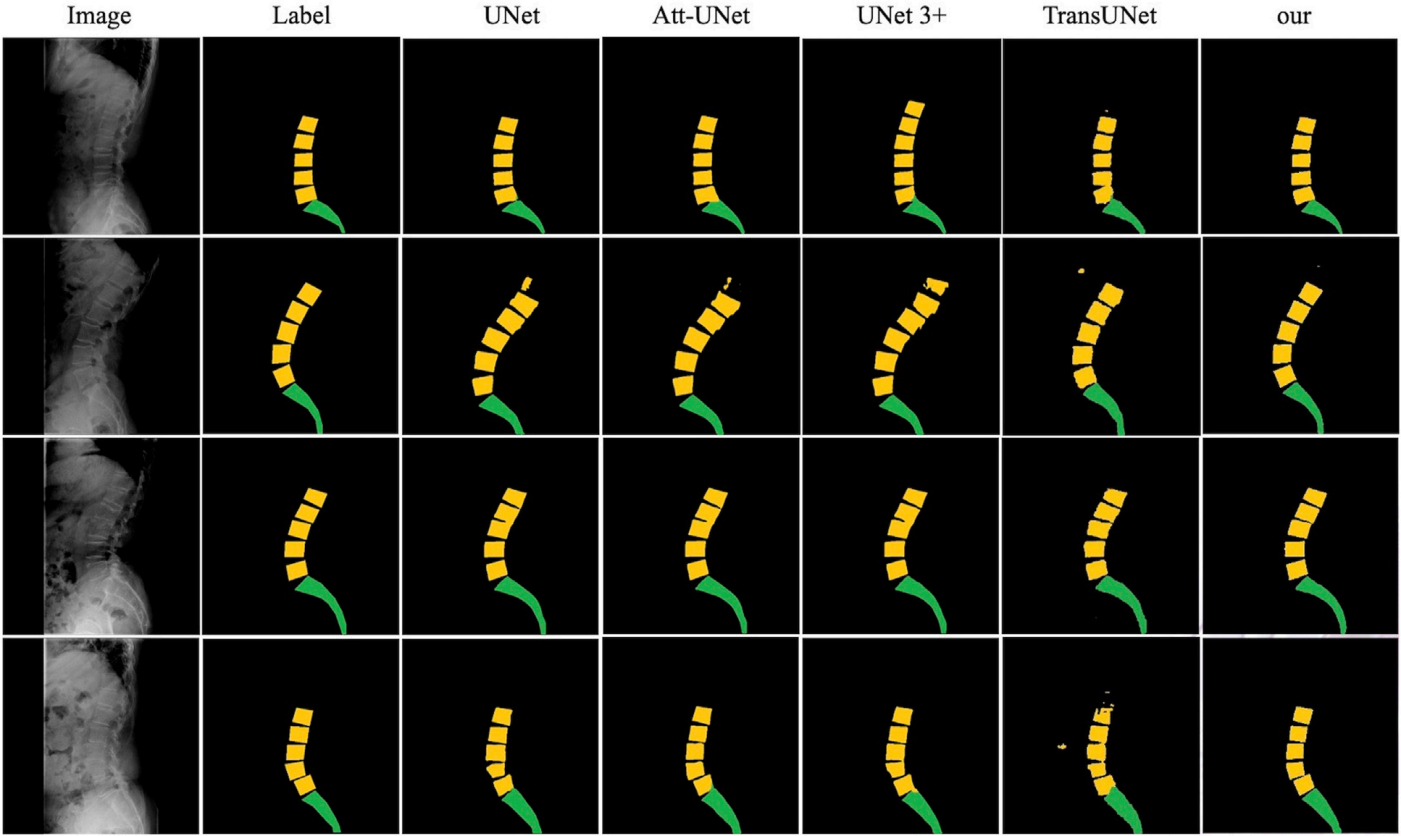
The comparison of the segmentation results of our model with UNet, Att-UNet, UNet 3+, and TransUNet.

For lumbar segmentation, the Dice coefficient and accuracy were 0.962 and 0.947, respectively. For sacrum segmentation, the Dice coefficient and accuracy were 0.954 and 0.939, respectively. The segmentation results for the lumbar region were better than those for the sacrum. Table 3 and Figure 6 showed that the segmentation performance of our model was better than the other four existing models.

**TABLE 3:** A comparison of the segmentation performance of our model with four other existing models.

Performance	UNet	Att-UNet	UNet 3+	TransUNet	Our
Lumbar					
Dice coefficient	0.937	0.946	0.947	0.937	0.962
Accuracy	0.925	0.943	0.939	0.942	0.947
Sacrum					
Dice coefficient	0.918	0.925	0.933	0.911	0.954
Accuracy	0.911	0.913	0.934	0.908	0.939

### 3.4 Performance of key point prediction

Table 4 shows that the PCK within the 3-mm distance threshold ranged from 88% to 98%.

**TABLE 4:** The PCK for key points at 1–5 mm distance thresholds.

Threshold(mm)	L1SA	L1SP	L4IA	L4IP	L5SA	L5SP	L5IA	L5IP	S1SA	S1SP
≤1	29	25	32	26	21	15	35	22	15	16
≤2	77	71	82	81	63	62	83	67	60	61
≤3	93	94	98	94	89	94	96	90	88	89
≤4	100	98	100	99	97	99	100	98	99	92
≤5	100	99	100	100	99	99	100	100	100	97

### 3.5 Measurement performance of the model

Comparing the measured values of the model with the reference standard, the result indicated that the reference standards for LL, SHA, ISA(L4–L5), ISA(L5–S1), PLS(L4–L5), PLS(L5–S1) were 49.77° ± 7.82°, 38.09° ± 6.85°, 14.86° ± 4.07°, 19.15° ± 8.07°, 12.27% ± 10.31%, and 13.36% ± 9.25%, and the measured values of the model were 49.20° ± 7.03°, 37.79° ± 6.53°, 14.15° ± 3.78°, 18.86° ± 7.63°, 12.45% ± 10.38%, and 13.58% ± 10.75%, respectively. The differences between them were not statistically significant (*p* > 0.05), as shown in Table 5.

**TABLE 5:** Measured values of three spine surgeons and the measured values of the model.

	S1	S2	S3	Mean	Model	t	*p*
LL (°)	49.82 ±7.79	49.65 ±7.84	49.90 ±7.91	49.77 ±7.82	49.20 ±7.03	0.58	0.58
SHA (°)	38.21 ±6.84	37.95 ±6.96	38.14 ±6.93	38.09 ±6.85	37.79 ±6.53	1.47	0.15
ISA(L4-L5) (°)	15.02 ±4.11	14.72 ±4.05	14.81 ±4.17	14.86 ±4.07	14.15 ±3.78	2.06	0.06
ISA(L5-S1) (°)	19.06 ±8.10	19.39 ±8.25	18.94 ±8.41	19.15 ±8.07	18.86 ±7.63	0.28	0.79
PLS(L4-L5) (%)	12.33 ±10.34	12.39 ±10.42	12.09 ±10.36	12.27 ±10.31	12.45 ±10.38	0.61	0.54
PLS(L5-S1) (%)	13.50 ±9.20	13.22 ±9.28	13.39 ±9.39	13.36 ±9.25	13.58 ±10.75	0.74	0.48

Data are expressed as mean ± SD.

*p* < 0.05 indicates that the difference between the measured values of the model and the reference standard is statistically significant.

**FIGURE 7:**
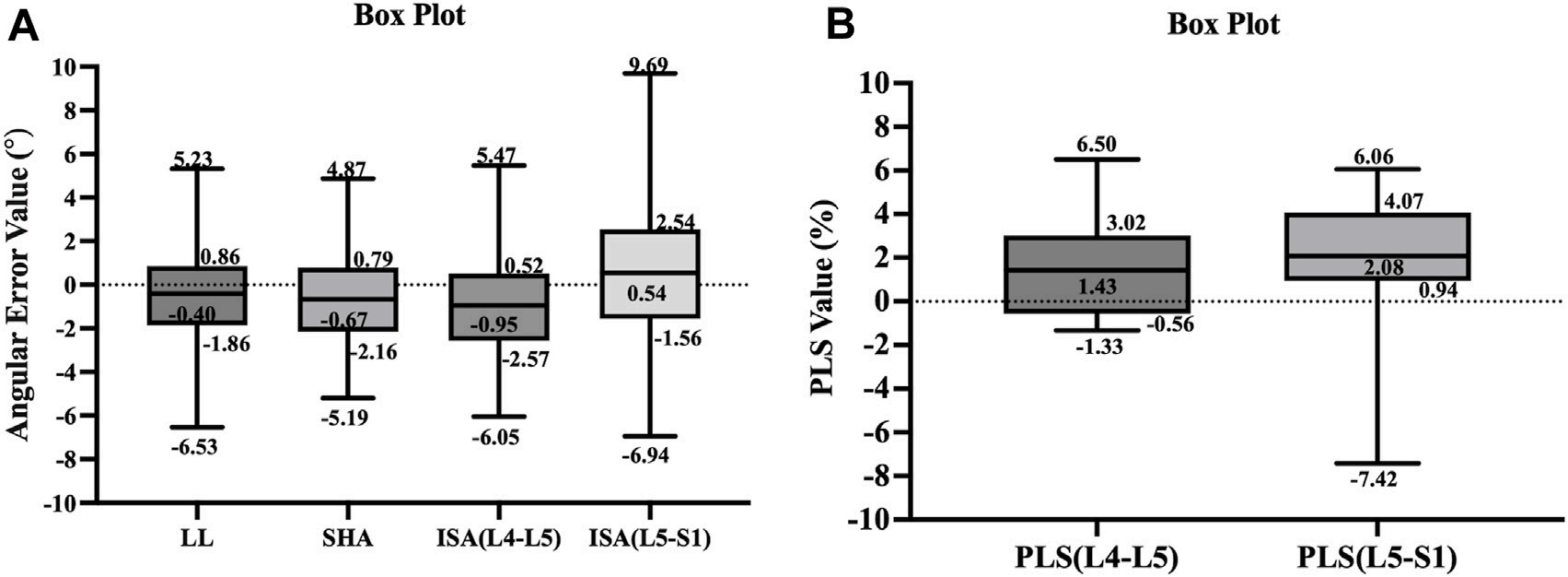
The box plots illustrate the distribution of error values between the measured values of the model and reference standard for the parameters of lumbar spine angle **(A)** and PLS **(B)**.

The results of our study found that the measured values of the model for lumbosacral radiographic parameters were consistent and reliable (LL, SHA, and ISA: ICC = 0.91–0.97, r = 0.91–0.96, MAE = 1.89–2.47, RMSE = 2.32–3.12; PLS: ICC = 0.90–0.92, r = 0.90–0.91, MAE = 1.95–2.93, RMSE = 2.52–3.70), as shown in Table 6. In addition, to visually demonstrate the robustness of the algorithm, statistical analyses were performed on the maximum, upper quartile, median, lower quartile, and minimum errors between the measured value of the model and the reference standard for the lumbar angular parameters and PLS, as illustrated in Figure 7.

**TABLE 6:** A comparison of the measured values of the model to the reference standard.

Parameter	ICC (95%CI)	r	MD	SD	MAE	RMSE
LL (°)	0.97(0.94–0.97)	0.96*	1.69	2.87	2.04	2.65
SHA (°)	0.94 (0.93–0.96)	0.94*	1.36	1.84	1.89	2.32
ISA(L4-L5) (°)	0.96 (0.95–0.97)	0.95*	1.55	2.41	2.08	2.59
ISA(L5-S1) (°)	0.91 (0.91–0.94)	0.91*	1.90	3.61	2.47	3.12
PLS(L4-L5) (%)	0.92 (0.89–0.92)	0.91*	1.60	0.24	1.95	2.52
PLS(L5-S1) (%)	0.90 (0.88–0.90)	0.90*	2.43	0.69	2.93	3.70

95% CI: 95% confidence interval.

*p < 0.05 indicates a statistically significant correlation between the reference standard and the measured values of the model.

## 4 Discussion

Accurate measurement of lumbosacral radiographic parameters of the lumbar spine is critical for biomechanics evaluation, clinical diagnosis, surgical planning, and prognosis prediction of lumbar diseases (Sparrey et al., 2014; Azimi et al., 2021; Schlösser et al., 2021). However, manual measurement of these parameters is time-consuming and laborious, and inevitably produces considerable variability. This study aimed to develop an accurate artificial intelligence automated measurement technique that could recognize and segment the lumbar and sacrum on lateral lumbar radiographs, as well as automatically measure lumbosacral radiographic parameters.

Our model had excellent segmentation quality and precise measurement of lumbosacral radiographic parameters. The Dice coefficient and accuracy of lumbar segmentation were 0.962 and 0.947, respectively. For sacrum segmentation, the Dice coefficient and accuracy were 0.954 and 0.939, respectively. Our study found that (Maher et al., 2017): The model accurately and automatically identified the key points, with the PCK ranging from 88% to 98% within the 3-mm distance thresholds (Kim et al., 2019); The ICC (MAE) for LL, SHA, and ISA ranged from 0.91 to 0.97 (1.89–2.47), and the ICC (MAE) for PLS ranged from 0.90 to 0.92 (1.95–2.93), which was comparable to or better than spine surgeons. The excellent performance of the model in measuring PLS is noteworthy. Due to the small volume of vertebrae, slight errors can cause significant changes in PLS values, leading to incorrect staging of lumbar spondylolisthesis. It is challenging to accurately quantify the severity of lumbar spondylolisthesis.

The traditional manual measurement method is based on the experience and judgment of the measurer, which results in inter-observer and intra-observer variability (Loder et al., 2004; Dang et al., 2005; Chan et al., 2014). Studies have shown that measurement errors can range from 3° to 10° (Loder et al., 2004; Mok et al., 2008). Chen et al. (2010) found that it was clinically significant to perform biomechanical or clinical analysis when the average distance between key points annotated by different observers was less than 3 mm. In this study, the percentage of key points annotated by different spine surgeons within the 3 mm threshold ranged from 81% to 83%, while the PCK predicted by the model within a 3 mm distance threshold ranged from 88% to 98%. This indicated that the measurement results of the model exceeded those of our spinal surgeons.

The segmentation network model developed in this study combined dilated convolution, RestNet, attention mechanism, multi-scale feature fusion, and other methods that offered high speed and high accuracy. We found that this model obtained these measurements much faster than spine surgeons, with the model obtaining measurements in 0.5 s instead of several minutes for spine surgeons. In addition, we used histogram enhancement, random Gamma transform, and random rotation on the images to increase data volume and improve the performance of the model. However, in the L5–S1 region, PCK predicted by the model within the 1–2 mm threshold was relatively poor due to the overlap of the iliac, lumbar, and sacrum on the lateral lumbar radiographs. This also explained why the segmentation results for the lumbar region were superior those for the sacrum. For the image overlap problem, we intend to manually adjust the results based on the prediction of the model and incorporate them into the training set to continuously optimize the algorithm.

Many studies had used deep learning-based models to automatically measure spinal parameters (Pang et al., 2019; Wang et al., 2019; Huang et al., 2020; Korez et al., 2020; Schwartz et al., 2021; Vrtovec and Ibragimov, 2022). Schwartz et al. (2021) used MultiResUNet for image segmentation and spinopelvic parameter calculations from lateral lumbar radiographs. The algorithm developed in their study worked well in segmenting images, with an overall Dice coefficient and an accuracy of 0.951 and 0.936, respectively. Referring to a systematic review published by Vrtovec and Ibragimov (2022), it was found that our model based on the RADMFNet algorithm outperformed models based on the UNet, Mask R-CNN and MultiResUNet algorithms in terms of segmentation performance. Korez et al. (2020) conducted a study using RetinaNet and U-Net algorithms to collect measurements in the sagittal plane. Their study included patients with internal spinal fixation devices; however, the proportion of images with internal fixation devices was not reported, and no subgroup analyses were performed to determine the effect of internal fixation devices on model performance. It is worth noting that most studies used annotations from a single or two observers (Pang et al., 2019; Wang et al., 2019; Huang et al., 2020; Korez et al., 2020; Vrtovec and Ibragimov, 2022), whereas our study used annotations from three observers, thus constructing a more reliable reference standard. To achieve high accuracy, it is essential to train the model on a large dataset. However, collecting and annotating images can be a time-consuming and expensive process (Willemink et al., 2020). In the future, while expanding the dataset, the training set can be enriched by using data enhancement (applying image flipping, panning, rotating, cropping, and intensity transformations), ensemble learning (training multiple models and then combining their results), or synthetic case generation (for example, using generative adversarial networks) (Shin et al., 2020; Vrtovec and Ibragimov, 2022).

Although this study has made advancements in automatically measuring lumbosacral radiographic parameters, some limitations remain. First, the training set for this study consisted of 992 lateral lumbar radiographs. For complex spinal diseases and clinical settings, this amount of data is insufficient. Furthermore, we included patients with an uneven age distribution and all from the same hospital, making it impossible to determine whether the performance of the model was influenced by age, X-ray machines from different hospitals, or variations in imaging acquisition techniques. In the future, we plan to use a larger, more diverse, and multicenter cohort to further train the model, increasing its clinical utility. Third, our model is currently unable to identify lumbosacral transitional vertebrae. One reason for this is that spine surgeons do not accurately identify and annotate lumbosacral transitional vertebrae. Another reason is that our algorithm has not yet incorporated the function to identify anatomical variations and abnormalities. Future studies could further include data from lumbosacral transitional vertebrae to determine whether the performance of the model is affected by both anatomical variations and abnormalities, allowing the model to be improved even further. Finally, a limitation of this algorithm is the error handling. The box plots demonstrated that the overall extreme error values of ISA and PLS were larger in the L5–S1 segment than those in the L4–L5 segment. Because in the fifth lumbar vertebra and sacral regions, segmentation defects may occur and the segmentation quality of the model was poor, leading to inaccurate measurements. We should incorporate error handling functionality into the algorithm to prevent the generation of highly erroneous measurement results when key points are not correctly identified. So far, such error handling capability has only been proposed in the study by Schwartz et al. (2021).

## 5 Conclusion

In summary, we had developed a model that could accurately identify vertebral key points and automatically calculate lumbosacral radiographic parameters. This model measured LL, SHA, ISA, and PLS on lumbar lateral radiographs with high accuracy and speed. Furthermore, compared to manual measurements, the measurements of the model were more consistent and reliable. The automatic measurement of lumbosacral radiographic parameters is anticipated to have a significant impact on spinal surgery in the coming years. In clinical practice, our model can help spinal surgeons save time and effort when measuring radiographic parameters. Besides, the model can be applied in research settings to conduct large-scale studies on lumbar anatomical parameters. In future research, we intend to include additional datasets and disease categories to improve the accuracy and stability of the model, as well as to continuously explore and improve the model’s algorithms.

## Data Availability

The raw data supporting the conclusions of this article will be made available by the authors, without undue reservation.
